# Sharing genetic test results of germline pathogenic variants of hereditary cancer with relatives: A single-center cross-sectional study

**DOI:** 10.1093/jjco/hyab110

**Published:** 2021-07-08

**Authors:** Naomi Fukuzaki, Yoshimi Kiyozumi, Satomi Higashigawa, Yasue Horiuchi, Maki Mizuguchi, Hiroyuki Matsubayashi, Seiichiro Nishimura, Keita Mori, Akifumi Notsu, Izumi Suishu, Sumiko Ohnami, Masatoshi Kusuhara, Ken Yamaguchi, Ardith Z Doorenbos, Yuko Takeda

**Affiliations:** Department of Nursing, Shizuoka Cancer Center Hospital, Shizuoka, Japan; Graduate School of Health Management, Keio University, Tokyo, Japan; Division of Genetic Medicine Promotion, Shizuoka Cancer Center, Shizuoka, Japan; Department of Nursing, Shizuoka Cancer Center Hospital, Shizuoka, Japan; Division of Genetic Medicine Promotion, Shizuoka Cancer Center, Shizuoka, Japan; Tokyo Metropolitan Institute of Medical Science, Tokyo, Japan; Division of Genetic Medicine Promotion, Shizuoka Cancer Center, Shizuoka, Japan; Division of Genetic Medicine Promotion, Shizuoka Cancer Center, Shizuoka, Japan; Division of Endoscopy, Shizuoka Cancer Center, Shizuoka, Japan; Division of Genetic Medicine Promotion, Shizuoka Cancer Center, Shizuoka, Japan; Division of Breast Surgery, Shizuoka Cancer Center Hospital, Shizuoka, Japan; Clinical Research Center, Shizuoka Cancer Center, Shizuoka, Japan; Clinical Research Center, Shizuoka Cancer Center, Shizuoka, Japan; Department of Nursing, Shizuoka Cancer Center Hospital, Shizuoka, Japan; Shizuoka Cancer Center Research Institute, Shizuoka, Japan; Shizuoka Cancer Center Research Institute, Shizuoka, Japan; Shizuoka Cancer Center, Shizuoka, Japan; Department of Biobehavioral Nursing Science, College of Nursing, University of Illinois Chicago, Chicago, USA; Graduate School of Health Management, Keio University, Tokyo, Japan

**Keywords:** family, genetic testing, germ-line mutation, hereditary, neoplastic syndromes

## Abstract

**Objective:**

This study aimed to determine whether Japanese cancer patients share test results of germline pathogenic variants of hereditary cancer with their relatives.

**Methods:**

This single-center cross-sectional study enrolled 21 Japanese patients who received results of germline pathogenic variants of hereditary cancer at least 6 months prior.

**Results:**

All patients shared their test results with at least one relative, with the following sharing rates: 85.7% for first-degree relatives, 10% for second-degree relatives and 8.3% for third-degree relatives. Patients most commonly shared the information with their children aged >18 years (86.7%), followed by their siblings (73.6%), spouses (64.7%) and parents (54.5%). Three categories were extracted from qualitative analysis: ‘characteristics of my cancer’, ‘knowledge and caution about inheritability’ and ‘utilization of medical care.’

**Conclusions:**

The rate of test result sharing with first-degree relatives was comparable with those in Europe and the USA. Patients with germline pathogenic variants also tended to share their test results more with their children and siblings than with their parents. Informing their relatives of the results was suggestive of the motivation to influence their relatives’ health outcome and contribute to the well-being of their children and siblings.

## Teaser

Japanese patients with germline pathogenic variants of hereditary cancers disclosed their test results to at least one relative, mostly first-degree relatives, including the patients’ adult children, siblings, spouses and parents.

## Introduction

Several studies have reported that 90–100% of individuals who receive genetic test results indicating germline variants of hereditary cancer (detected by single-gene and exome sequencing) share their test results with at least one immediate family member (1–5). In contrast, another study (6) concluded that the effect and value of providing personal results of whole-genome sequencing remained unclear.

According to American Society of Clinical Oncology guidelines (2020) (7), first- or second-degree blood relatives of patients with ovarian cancer with known germline pathogenic cancer susceptibility gene variants should be offered individualized genetic risk evaluation, counseling, and genetic testing. However, only a few studies worldwide have investigated whether cancer patients with germline pathogenic variants of hereditary cancer share their genetic test results with their relatives.

In Japan, 75% of individuals who met the clinical criteria for Lynch syndrome advised their relatives to seek a medical assessment (8). On the other hand, Asians are reported to have a lower rate of disclosure of BRCA1/2 genetic test results to family members than Westerners (9,10). Moreover, the actual status of genetic test result disclosure to relatives in the Japanese population remains unclear. To our knowledge, this is the first study to investigate how Japanese cancer patients with hereditary germline pathogenic variants share their test results to their family members. This study could serve as a reference for patients and an educational tool for medical professionals in cancer genomic medicine in Japan.

This study included Japanese patients with germline pathological variants of hereditary cancer. We aimed to determine the rate patients share test results with relatives (first-, second- and third-degree blood relatives and spouses or partners). In addition, the study aimed to investigate the content of the information that was shared.

## Patients and methods

### Study setting

Ethical approval for this study was obtained from the Institutional Review Board of the Shizuoka Cancer Center (SCC) hospital (No. 30–33–30-1-7) and the Keio University Graduate School of Health Management (No. 2019–06). Written informed consent was obtained from each participant. This study was carried out in accordance with the 1964 Declaration of Helsinki and its later amendments.

In April 2020, The SCC was designated as a core center for cancer genome medicine in Japan. This cross-sectional study was conducted at SCC from May 2019 to March 2020. The participants were cancer patients who were clinically diagnosed with hereditary cancers with germline pathogenic variants between January 2014 and March 2020. The disclosure of results about germline pathogenic variants at the SCC was conducted in face-to-face genetic counseling sessions, in collaboration with the physician in charge, clinical geneticists, certified genetic counselors, and nurses, after confirming the patient’s psychological situation and their willingness to receive the results. When disclosing the results, the genetic counselors explain the importance of sharing the results with relatives and the problems involved, and patients were allowed to decide whether to share the information with relatives, the details to share and the timing. Patients who stated that they would find it difficult to explain the results to their relatives, were informed that the genetic counselor could explain the results to their relatives in person or via phone on request.

### Eligibility criteria for study participants

The inclusion criteria were as follows: (1) patients aged ≥20 years with a cancer diagnosis, as outlined above; (2) patients who were informed about their genetic test results at least 6 months previously. The 6-month period was chosen because in previous studies (11–13), it was concluded that psychological distress caused by the knowledge of genetic test results increases immediately after the receival of the results and decreases with the passage of time, over a period of approximately 6 months; (3) patients with a current Eastern Cooperative Oncology Group performance status of 0–2, (4) those with no history of psychiatric illness and (5) patients who received permission from a clinical geneticist. The exclusion criteria were as follows: (1) patients whose family members had undergone gene testing, (2) patients who were unable to understand or speak Japanese, (3) those who were undergoing treatment or follow-up at another hospital, and (4) patients with no living relatives. Patients who had a blood relative that had received a cancer diagnosis and test results indicating germline variants were excluded because the presence of germline variants was already shared with someone in the family; therefore, they were ineligible.

### Data collection

Clinical geneticists and genetic counselors selected the participants based on the inclusion and exclusion criteria. Next, genetic counselors explained the outline of the study to patients who visited the outpatient clinic during the study period. Patients who listened to the detailed explanation were given another verbal and written explanation in a private room, and those who signed the consent form were enrolled in the study. Considering the confidentiality of the test results and the psychological burden, the ethics review committee of our institution instructed us not to audio-record our conversations with the patients. Therefore, the genetic counselors obtained qualitative data by making notes on patients’ response during the conversations. Patients completed self-administered questionnaires about their demographic and clinical characteristics, and were then interviewed by genetic counselors. The interview was based on a questionnaire that was created independently by the authors, which included both closed-ended and open-ended questions. Answering the questions was optional.

#### Demographic and clinical characteristics

Patients responded to questions regarding their educational level, marital status, and cancer treatment history. Other information was collected from the patients’ medical records, including age, sex, disease, type and date of surgery, types of germline pathogenic variants, family tree, family history of cancer, and date of result disclosure.

#### Sharing results with family members

The genetic counselors asked the patients if they had shared their test results with anyone. If they answered ‘yes’, they were asked about their relationship with the person and their age and place of residence (0: lives with the patient, 1: lives in the city, 2: lives outside the prefecture, 3: lives outside the country). Parents, children, and siblings were classified as first-degree relatives; grandparents, uncles/aunts, nephews/nieces, and grandchildren were classified as second-degree relatives; and great-grandparents, great-uncles/aunts, cousins, children of nephews/nieces, and great-grandchildren were classified as third-degree relatives. Spouses, former spouses, and partners, including unmarried partners, were categorized as spouses. From medical records, each patient’s family tree, up to third-degree relatives, was checked. If relatives were alive, a value of one was assigned; otherwise, zero was assigned. The denominator was the total number of relatives (each counting as 1) for each patient. If patients shared their results with a particular type of family member, a value of one was assigned; otherwise, zero was assigned (14,15). The total number of family members with whom the patient shared their results was used as the numerator, and the sharing rate was calculated. Patients who had not shared their results with their first-degree relatives were asked about the reason for not sharing their refusal.

#### Details of the shared information

We asked the patients ‘What did you share with your relatives? Is there anything you intentionally did not share?’ Patients were allowed to answer these questions freely. The genetic counselors recorded patients’ responses as bullet points or short sentences. The information was considered qualitative data.

### Data analysis

To calculate totals, means, medians, standard deviations, and 95% confidence intervals (CIs), quantitative data were tabulated using R statistical package, version 4.0.3 (The R Foundation; Vienna, Austria). Qualitative data were evaluated using content analysis (16–18), as per the following steps, with reference to previous studies (19). Information from interviews was used as text input, which was divided into units and condensed, whilst preserving the core meaning. The condensed units were labeled by formulated codes. Subsequently, categories were formed by grouping codes that were related in content or context. To ensure data integrity during the analysis, we received guidance from expert researchers in cancer nursing and qualitative research.

## Results

### Demographic and clinical characteristics

From January 2014 to March 2020, 66 patients were diagnosed with germline pathogenic variants of hereditary cancer at the SCC, and 65 of them received the results. The patient who did not receive the results considered receiving the results unnecessary because he had no living relatives. Based on the inclusion and exclusion criteria, 22 patients were excluded from the study for the following reasons: positive diagnosis in blood relatives (*n* = 12), death (*n* = 5), ongoing treatment in other hospitals (*n* = 2), and less than 6 months since the receival of test results (*n* = 3). Of the remaining 43 participants, 21 (49%) consented to participate in the study. The other candidates did not participate in the study because they either did not meet the inclusion criteria or chose not to participate. Six patients (29%) were male, and 15 (71%) were female; the median (interquartile range) age was 56 (28–79) years. Sixteen (76%) patients were married, and 14 (66%) were high-school graduates. Eleven patients had hereditary breast and ovarian cancer, six had Lynch syndrome, two had familial adenomatous polyposis, one had multiple endocrine neoplasia type 1, and one had multiple endocrine neoplasia type 2. All 21 patients had undergone at least one surgery. Seventeen (80%) patients were receiving postoperative follow-up. Twenty (95%) patients had at least one other family member with cancer. Eighteen patients (86%) were living with family members, such as their spouse or children, and three patients (14%) were living with their parents. The median (interquartile range) time between when the patients disclosed their results and when this investigation was conducted was 777 (194–1472) days. Other patient characteristics are presented in Table 1.

**Table 1:** Characteristics of patients (*n* = 21)

Characteristic	Variable	*n* (%)
Sex	Men	6 (29)
	Women	15 (71)
Median age & range (years)		56 (28–79)
Age (years)	20–29	1 (5)
	30–39	3 (14)
	40–49	4 (19)
	50–59	4 (19)
	60–69	7 (33)
	70+	2 (10)
Marital status	Married	16 (76)
	Single	2 (10)
	Widowed	2 (10)
	Divorced	1 (4)
Education	≤High school	14 (66)
	>High school	7 (34)
Diagnosis	Hereditary breast and ovarian cancer	11 (52)
	Lynch syndrome	6 (29)
	Familial adenomatous polyposis	2 (10)
	Multiple endocrine neoplasia type 1	1 (4)
	Multiple endocrine neoplasia type 2	1 (4)
Operation history	Yes	21 (100)
Current treatment	Postoperative follow-up	17 (80)
	Recurrence chemotherapy	2 (10)
	Endocrine therapy	2 (10)
Family history of cancer	Yes	20 (95)
	No	1 (5)
Family member living with the patient	Spouse and children	10 (48)
	Spouse only	6 (28)
	Children only	2 (10)
	Parents	3 (14)
Median & range of time elapsed between result disclosure and investigation (days)		777 (194–1472)

Categorical variables are presented as proportions (percentage) and continuous variables as medians (interquartile range).

### Sharing results with family members and reasons for not sharing results

All patients had relatives up to the third degree of consanguinity, and the number of relatives with whom the patients shared their results ranged from 1 to 7. All patients shared their results with at least one relative (Table 2); all relatives with whom results were shared were 18 years or older. Eighteen of 21 patients (85.7%, 95% CI 64–97%) shared their results with a blood relative, and three patients (14.3%, 95% CI 0–29%) did not share the results with their blood relatives, but shared them with their spouses. Eighteen of 21 patients (85.7%, 95% CI 64–97%) shared their results with at least one family member who was living with them.

**Table 2:** Sharing rate of germline pathogenic variant results by family member type

Type of family members by patients	No. of patients with family members, *n*	No. of patients who shared, *n* (%)	95% CI, %^c^
Blood relatives and spouse (all) (age ≥ 18 years)	21	21 (100.0)	84–100
Blood relatives	21	18 (85.7)	64–97
Spouse	21	3 (30.0)	7–65
First-degree relatives (all)	21	18 (85.7)	64–97
Parents	11	6 (54.5)	23–83
Father^a^	10	3 (30.0)	7–65
Mother^a^	9	5 (55.6)	21–86
Children (all)	19	13 (68.4)	43–87
Sons	15	9 (60.0)	32–84
Daughters	17	10 (58.5)	33–81
Children (age ≥ 18 years)	15	13 (86.7)	60–98
Sons	11	9 (81.8)	48–98
Daughters	12	10 (83.3)	52–98
Children (age < 18 years)	6	0	0
Siblings	19	14 (73.6)	49–91
Brothers^a^	12	8 (66.7)	35–90
Sisters^a^	14	8 (57.1)	29–82
Second-degree relatives (all)	20	2 (10.0)	12–32
Aunt^a^	15	1 (6.7)^b^	0–31
Niece^a^	13	1 (7.7)	0–36
Third-degree relatives^a^	12	1 (8.3)	0–38
A female first cousin^a^	6	1 (16.7)^b^	0–64
Spouse^a^	17	11 (64.7)	38–86
At least one family member living with the participant	21	18 (85.7)	64–97

^a^All participants were aged ≥18 years.

^b^Same patient.

^c^95% confidence interval (CI) of sharing rate.

Among first-degree relatives, 11 patients had at least one living parents, with whom six (54.5%, 95% CI 23–83%) patients had shared their results. Nineteen patients had children, including children aged <18 years, with whom 13 (68.4%, 95% CI 43–87%) shared their results. Fifteen patients had children aged >18 years, and 13 shared their results with them (86.7%, 95% CI 60–98%). None of the patients shared their results with children aged under 18 years. Nineteen patients had siblings (aged >18 years), with whom 14 shared their results (73.6%, 95% CI 49–91%).

In terms of relationships, the results were most shared with daughters aged ≥18 years; 10 (83.3%, 95% CI 52–98%) of 12 patients shared their results with daughters aged ≥18 years. This was followed by sons aged ≥18 years in 9 of 11 patients (81.8%, 95% CI 48–98%). The third most common relationship was sibling, especially brothers (66.7%, 95% CI 35–90%), followed by sisters (57.1%, 95% CI 29–82%). Moreover, results were shared with mothers (55.6%, 95% CI 21–86%) and fathers (30%, 95% CI 7–65%).

Eight (38%, 95% CI 18–61%) patients shared their results with all surviving first-degree relatives, and the remaining 13 patients (62%, 95% CI 38–82%) did not share the information with some first-degree relatives. The patients did not inform first-degree relatives about their results for reasons such as differences in age, unfamiliarity, differences in their sex, or because relatives were unmarried status or did not have children. Most participants were particularly reluctant to inform their parents because they did not want them to worry.

Of 20 patients with second-degree relatives, two (10%, 95% CI 12–32%) patients disclosed their results to them (an aunt and a niece). Of 12 patients with third-degree relatives, one (8.3%, 95% CI 0–38%) patient shared the results with a third-degree relative (a female cousin). All patients who disclosed their results to second- and third-degree relatives were women. Of 17 patients with spouses, 11 (64.7%, 95% CI 38–86%) patients shared their results with their spouses.

### Shared and unshared information

Details of shared and unshared information are presented in Table 3. Of the 21 patients, two patients were excluded because they did not specify what information they had shared with their relatives or withheld, giving answers such as: ‘I told them the results’, in the free-text field. Regarding shared information, 69 statements were obtained from 19 patients, and the information was classified into three categories (‘characteristics of my cancer’, ‘knowledge and caution about inheritability’ and ‘utilization of medical care’) and 10 codes (<hereditary cancer>, <disease or gene name>, <origin such as paternal or maternal>, <possibility of passing down the condition>, <susceptible cancer types>, <50% of probability of inheritance>, <effects on nephews and nieces>, <need for gene testing of relatives>, <recommendations for clinical laboratory tests>, and < places to consult>). The topic that was shared by most patients was <need for gene testing of relatives>.

**Table 3:** Shared and unshared contents (multiple answers)

Categories	Codes (*n*)
Shared contents *n* = 19	
Characteristics of my cancer	Hereditary cancer (12)
	Disease or gene name (5)
	Paternal, maternal, etc., origin (2)
Knowledge and caution to inheritance	Possibility of passing down the condition (14)
	Susceptible cancer types (6)
	50% of probability of inheritance (4)
	Effects on nephews and nieces (2)
Utilization of medical care	Need for gene testing in relatives (18)
	Recommendations for clinical laboratory tests (5)
	Places to consult (1)
Unshared contents *n* = 3	
Effects on second-degree relatives	Effects on nephews and nieces (3)

Regarding unshared information, three patients were explicit about what was not shared. However, as the other 16 patients did not provide details about unshared information, we assumed that they had no unshared information. The chosen category was ‘effects on second-degree relatives’, and the code was <effects on nephews and nieces>. The three patients who reported unshared contents had shared their results with relatives for reasons such as <possibility of passing down the condition> and < need for gene testing of relatives>.

## Discussion

The findings showed that all patients shared their results with at least one relative, which was most often a first-degree relative, especially children over 18 years of age. The relative with whom information was least shared with was parents. The most common shared code was ‘need for gene testing of relatives’, one of the derived codes, ‘effects on nephews and nieces’, was found in both the shared and unshared information.

Consistent with previous studies that included Western populations, we found that all our Japanese patients shared their results with at least one relative and 85.7% of the patients shared their results with first-degree relatives (9,20–23). Other studies on individuals with germline pathogenic variants of hereditary cancer (1,2) have reported that over 90% of the patients shared their results with their parents; however, in our study, only 54.5% of the participants shared their results with their parents. The decision to share results with parents may be influenced by the age of the parents, concerns about the psychological effect the results may have on them, a lack of intimacy, and distance. Studies that have examined the reasons for patients from Western countries not sharing their genetic test results (2,23) have revealed reasons such as lack of a close relationship, unfamiliarity, belief that the relative is aware of the genetic situation, and avoidance of the negative emotional effect of the information. The same reasons seem to apply to Japanese cancer patients. Additionally, in this survey, three patients were living with their parents, while the other patients were living in a nuclear family setup. It should be noted that similar results may not have been obtained if the majority of patients had been living alone or in a multi-generational household.

Second, the reason why a high proportion of the participants shared their results with their children and siblings may be attributed to the notion that the information would contribute to the wellbeing of the relatives. This abovementioned notion is supported by the information the patients shared with these family members. Patients were found to have shared ‘characteristics of my cancer’, ‘knowledge and caution about inheritability’ and ‘utilization of medical care.’ Asian patients with germline pathogenic variants reported (24) a strong motivation to share their results with their family members, which was connected to the intention to influence disease outcome in their relatives, since a timely warning would allow their relatives take necessary precautions. Similarly, we believe that patients in our study may have shared more information on the benefits of preventive measures with their children and siblings, who could benefit from this information, given that the commonly shared information were ‘knowledge and caution about inheritability’ and ‘utilization of medical care.’

Finally, the proportion of participants who shared their results with second- and third-degree relatives was lower than that shared with first-degree relatives. We believe this may be due to reasons such as ‘effects on nephews and nieces’, which was a common issue in both the shared and unshared information. The parents of the patients’ nephews and nieces were the patients’ siblings; thus, the patients may have not told their nephews and nieces because they expected their parents to tell them. A previous study (2) also reported that when genetic information was passed from the originator to more distant relatives, it was most often passed from a first-degree relative to a first-degree relative. The patients in the present study who shared ‘effects on nephews and nieces’ seem to fall into this category, suggesting that this is one of the reasons why the rate of sharing from patients to second-degree relatives was low, and that even if the information is not shared directly by patients, it may be shared by first-degree relatives. Conceivably, by informing the patient’s siblings about the hereditary variant and the possible effect on the family tree, these siblings were expected to inform their children. However, the only unshared content was ‘effects on nephews and nieces.’ Lafrenière et al. (25) reported that result disclosure was motivated by the individual’s ‘personal characteristics’ and non-disclosure was motivated by the ‘personal characteristics of family members.’ In this study, patients who did not share information, based on the ‘effects on nephews and nieces’, shared other contents to their relatives. This indicates that a negative situation may have arisen during the process of ‘sharing’ or the reaction of other people may have made the patients decide against sharing the information. Since individual factors are considered to influence ‘sharing’, we think it is necessary to explore the process of ‘sharing’ in future studies, with considerations of the backgrounds of patients and their relatives.

We expect that the results of this study will be widely applied to clinical practice in the future. The results of this study could assist decision-making in patients who are considering sharing their genetic test results. We are confident that nurses, especially Advanced Practice Nurses (such as Certified Nurse Specialists in Genetics Nursing or Cancer Nursing as defined by Japanese Nursing Association) possess specialized knowledge and complex decision-making skills. In addition, it is desirable to establish a prevention system for relatives, as the study found that patients were motivated to share their test results to contribute to the health outcomes among their relatives.

This study has several limitations. First, it was a cross-sectional study conducted at a single center in a local city with a small sample size. The study design limits the generalizability of the results. Specifically, as the survey was conducted in a small city, the number of people living alone tended to be smaller than in large cities. Moreover, although the genetic counselors strove to avoid influencing the will of the patients and provide explanations, the way explanations were provided may have inadvertently affected the patients’ choices to share information and the information that was shared. We were unable to identify the factors that affected information sharing in this study, and this requires further research In addition, the sharing of genetic information with relatives is not compulsory. If we repeatedly ask patients why they have not shared their test results, they would feel obligated to share the results. Therefore, we limited ourselves to asking only the first-degree next of kin why they did not share. Furthermore, due to the confidential nature of genetic information, we did not record the interviews, and this limits the generalizability of the qualitative data. Second, only those with a performance status score of 0–2 and patients with stable mental status were selected. This selection criteria may have introduced selection bias. In addition, Twenty-one of 43 patients (49%) who met the eligibility criteria were included in this study. They might have been eager to participate because they were satisfied with the process of obtaining the results of genetic testing and sharing the findings. Finally, there may have been some recall bias since patients were asked to recall events that occurred at least 6 months earlier. Future studies should include longitudinal surveys on both patients and their relatives, which would allow the extraction of more accurate results while considering the psychological effect on the patients.

## Conflict of interest statement

Ken Yamaguchi received royalties from the Pro GRP patent. All other authors have no conflict of interest to declare.

## Funding

The authors did not receive any funding for this study.

## Data sharing and data accessibility

Data will be shared upon reasonable request to the corresponding author.
